# High hydrostatic pressure upregulate central carbon metabolism genes in a distillery yeast strain

**DOI:** 10.1186/1753-6561-8-S4-P207

**Published:** 2014-10-01

**Authors:** Mainã Mantovanelli Mota, Fernanda Bravim, Jimmy Soares, Tássia Nati, James Riley Broach, Antonio Alberto Ribeiro Fernandes, Patricia Machado Bueno Fernandes

**Affiliations:** 1 Universidade Federal do Espírito Santo, Vitória, ES, Brasil; 2 Penn State University College of Medicine, Hershey, PA 17033, USA

## Background

High hydrostatic pressure (HHP) is applied on a variety of biotechnological process, including food preservation, modulation of enzymatic activity, disaggregation of proteins, vaccines development and recently it was reported its use for ethanol yield increase [1,2].

## Methods

In this study we performed a microarray analysis in a distillery *Saccharomyces cerevisiae *strain (BT0510) submitted to sublethal pressure treatment of 50 MPa for 30 min at room temperature, followed by incubation for 5, 10 and 15 min at room pressure (0.1 MPa). The transcription of the genes involved in central carbon metabolism in response to HHP was investigated for bioinformatics tools.

## Results

HHP induced genes related to the phosphorylation of intracellular glucose, *HXK1 *and *GLK1*. Glucose-6-phosphate is a glycolysis and pentose phosphate pathway (PPP) intermediary. Glycolysis and gluconeogenesis were slightly induced by pressure, but *GPD1 *and *GUT2*, associated with the glycerol-3-phosphate shuttle, were upregulated by HHP. This mechanism transfer reducing equivalents from the cytosol to the mitochondria for the reduction of ubiquinone in ubiquinol in the electron transport chain that leads to oxidative stress. Interesting enough, HHP also induced *ZWF1 *and *GND2 *genes that encode two enzymes related to NADPH production in PPP. The PPP plays an important role in NADPH generation, which is required in oxidative stress response, in order to maintain the intracellular levels of reduced glutathione [3,4]. Pyruvate, product of glycolysis in cytosol, is converted to different metabolites that are transported to the mitochondria and integrate the tricarboxylic acid cycle (TCA cycle). The HHP upregulated several genes associated to this pathway, such as *LPD1*, related to the conversion of pyruvate into acetyl-CoA in the mitochondria, and *PDC1 *and *PDC6 *correlated with the conversion of cytosolic pyruvate in acetaldehyde. Acetaldehyde is converted in acetate in the mitochondria by Ald4p and, subsequently, in acetyl-CoA by Acs1p and directed do TCA cycle; genes that encode these enzymes were also induced by HHP. Fatty acid biosynthesis related genes were not strongly affected by HHP. This evidence reinforces the hypothesis that acetyl-CoA is directed to the TCA cycle after HHP stress. HHP also upregulated the glutamate degradation I pathway genes (*GAD1, UGA1 *and *UGA2*). This pathway plays an important role in oxidative stress, and produces NADPH and succinate, which is transported into the mitochondria and participate in the TCA cycle [5]. The induction by HHP of the TCA cycle genes and of many genes of the electron transport chain are a strong evidence that yeast modulate its metabolism after stress in order to increase respiration. However, genes involved in fermentation (*PDC1, PDC6, ADH1 *e *ADH5*) were also upregulated, suggesting that anaerobic metabolism was not completely repressed during HHP stress and during the recuperation period.

## Conclusions

Our data showed that several genes related to the central carbon metabolism were induced by HHP, suggesting that yeast accelerates glucose consumption and, consequently, its metabolism. This data analysis allows the design of new cells for ethanol yield increase using HHP.

## Acnowledgements

CAPES, CNPq, FINEP, FAPES and MCT.
